# Implementation of Remote Activity Sensing to Support a Rehabilitation Aftercare Program: Observational Mixed Methods Study With Patients and Health Care Professionals

**DOI:** 10.2196/50729

**Published:** 2023-12-08

**Authors:** Ziyuan Lu, Tabea Signer, Ramona Sylvester, Roman Gonzenbach, Viktor von Wyl, Christina Haag

**Affiliations:** 1 Institute for Implementation Science in Health Care University of Zurich Zurich Switzerland; 2 Valens Rehabilitation Centre Valens Switzerland; 3 Epidemiology, Biostatistics and Prevention Institute University of Zurich Zurich Switzerland

**Keywords:** physical activity, activity sensor, normalization process theory, rehabilitation, chronic disease, chronic, aftercare, sensor, sensors, exercise, neurology, neuroscience, neurorehabilitation, adherence, need, needs, experience, experiences, questionnaire, questionnaires, mobile phone

## Abstract

**Background:**

Physical activity is central to maintaining the quality of life for patients with complex chronic conditions and is thus at the core of neurorehabilitation. However, maintaining activity improvements in daily life is challenging. The novel Stay With It program aims to promote physical activity after neurorehabilitation by cultivating self-monitoring skills and habits.

**Objective:**

We examined the implementation of the Stay With It program at the Valens Rehabilitation Centre in Switzerland using the normalization process theory framework, focusing on 3 research aims. We aimed to examine the challenges and facilitators of program implementation from the perspectives of patients and health care professionals. We aimed to evaluate the potential of activity sensors to support program implementation and patient acceptance. Finally, we aimed to evaluate patients’ engagement in physical activity after rehabilitation, patients’ self-reported achievement of home activity goals, and factors influencing physical activity.

**Methods:**

Patients were enrolled if they had a disease that was either chronic or at risk for chronicity and participated in the Stay With It program. Patients were assessed at baseline, the end of rehabilitation, and a 3-month follow-up. The health care professionals designated to deliver the program were surveyed before and after program implementation. We used a mixed methods approach combining standardized questionnaires, activity-sensing data (patients only), and free-text questions.

**Results:**

This study included 23 patients and 13 health care professionals. The diverse needs of patients and organizational hurdles were major challenges to program implementation. Patients’ intrinsic motivation and health care professionals’ commitment to refining the program emerged as key facilitators. Both groups recognized the value of activity sensors in supporting program implementation and sustainability. Although patients appreciated the sensor’s ability to monitor, motivate, and quantify activity, health care professionals saw the sensor as a motivational tool but expressed concerns about technical difficulties and potential inaccuracies. Physical activity levels after patients returned home varied considerably, both within and between individuals. The self-reported achievement of activity goals at home also varied, in part because of vague definitions. Common barriers to maintaining activity at home were declining health and fatigue often resulting from heat and pain. At the 3-month follow-up, 35% (8/23) of the patients withdrew from the study, with most citing deteriorating physical health as the reason and that monitoring and discussing their low activity would negatively affect their mental health.

**Conclusions:**

Integrating aftercare programs like Stay With It into routine care is vital for maintaining physical activity postrehabilitation. Although activity trackers show promise in promoting motivation through monitoring, they may lead to frustration during health declines. Their acceptability may also be influenced by an individual’s health status, habits, and technical skills. Our study highlights the importance of considering health care professionals’ perspectives when integrating new interventions into routine care.

## Introduction

### Background

Patients attending inpatient neurorehabilitation often face complex chronic health conditions, such as multiple sclerosis, or cardiovascular diseases, such as stroke [1,2]. These chronic health conditions are typically linked to increasing physical impairments, which negatively impact individuals’ quality of life [3,4]. They require complex treatment and care, often involving multiple, interdisciplinary health care providers and a blend of pharmacological treatments, physical therapy, and lifestyle management [5-7].

Over the recent years, the promotion of physical activity has become state of the art in inpatient rehabilitation treatment for chronic diseases or diseases with risk for chronicity [8-14]. However, any improvements in daily activity levels and physical fitness that may be achieved in a rehabilitation setting are typically challenging to maintain in daily life [15-17]. Once back in their daily-life environment, persons with chronic diseases face multiple barriers to physical activity, such as fatigue; a less structured environment; or time restrictions associated with care responsibilities, employment, and other factors [18-23]. The Valens Rehabilitation Centre has recently developed a novel inpatient routine aftercare program, designed to prepare patients for this transition, named the Stay With It program (Swiss German: Bliib dra program). Complementing inpatient rehabilitation treatment, it combines motivational interviewing techniques with detailed action plans for promoting physical activity following rehabilitation.

The emergence of novel activity sensors designed for daily-life physical activity tracking has created novel avenues for promoting self-monitoring and self-management skills, and these sensors are well suited to support the integration of modules into patients’ daily lives [19,20,24-26]. Many consumer-grade activity sensors assess a broad range of activity- and sleep-related parameters in real time, including step count, activity levels, heart rate (variability), and sleep stages. A previous umbrella meta-analysis found good evidence for the beneficial health effects of activity sensor–supported lifestyle management changes, particularly for physical activity outcomes or weight loss [27]. Conventional aftercare programs for rehabilitation, such as the Stay With It program, may, therefore, benefit from the additional systematic integration of activity sensors into their educational and aftercare monitoring procedures. Indeed, a previous study conducted in the same setting in Valens with people with multiple sclerosis suggested that many patients perceived activity sensors as devices that help maintain motivation for physical activity after discharge at home [28]. Although these novel technical innovations have potential in complementing already established routine care programs, their implementation faces multiple challenges. Potential challenges include the time required for the ongoing resolution of device-related technical issues and the commitment required to guide patients in understanding their data [26,28].

Planning the integration of a digitally supported aftercare program, such as Stay With It, into clinical routine care is a complex process, requiring the involvement of a broad range of stakeholders and the adaptation of their standard workflows. Many aspects of the planning, implementation, and evaluation of such digitally augmented programs would clearly benefit from guidance from different implementation frameworks [29]. In the context of digitally supported interventions, the normalization process theory (NPT) is a well-established framework for guiding the implementation of novel interventions in routine settings [30,31]. The NPT consists of 4 core constructs, namely “coherence,” “cognitive participation,” “collective action,” and” “reflexive monitoring,” which represent distinct steps needed in the implementation process, referred to as “normalization” [32]. The definitions of the NPT constructs and their application and relevance to both patients and health care professionals in our study are presented in Table 1.

However, there currently exists no specific guidance on the implementation of digital tools into the workflows of an established, conventional motivational program. For example, how receptive will health care providers be about the integration of activity sensors? What needs for educational support or administrative changes may arise from the add-on implementation, both for patients and health care providers?

**Table 1:** The 4 constructs of the normalization process theory (NPT) and how they apply to this study.

Description of NPT construct	Patients	Health care professionals
“Coherence” refers to the sense-making process at the individual and group levels [32,33].	With respect to patients, “coherence” refers to their understanding of the purpose of the program and how it, possibly in combination with an activity sensor, could support them in staying active at home.	“Coherence,” as it relates to the health care professionals in this study, refers to their understanding of the purpose of the program and how an activity sensor can support implementation.
“Cognitive participation” refers to the relational work of forming and maintaining a practice community around a complex intervention or novel technology [32,33].	With respect to patients, “cognitive participation” refers to adapting daily routines to incorporate the program and related activities as well as understanding how the activity sensor can be used in and best integrated into daily life, which may involve resolving technical issues by acquiring new technical skills.	With respect to the health care professionals in this study, “cognitive participation” refers to ensuring the necessary knowledge of the program and associated procedures and collaborating as a team to facilitate implementation. Throughout program implementation, this is an ongoing process.
“Collective action” refers to the operational work required to implement new practices related to new technologies or complex interventions [32,33].	With respect to patients, “collective action” means creating an environment and circumstances that are conducive for them to benefit from the program. This may include, for example, shifting priorities or changing personal routines.	With respect to health care professionals, “collective action” means taking care of organizational tasks to facilitate program implementation and seamlessly integrate the new program and its activity sensor into their daily tasks, ensuring efficiency while minimizing their time commitment to an appropriate level.
“Reflexive monitoring” pertains to the evaluative work of assessing and understanding how a practice affects individuals themselves and their surroundings [32,33].	Reflexive monitoring, as it relates to the patients in this study, concerns how they evaluate the way in which the novel program and, if applicable, its combination with an activity sensor affect them (eg, health and daily life) and their environment (eg, family and responsibilities).	In this study, health care professionals who engaged in reflective monitoring evaluated the impact and effectiveness of the new program, especially when paired with an activity sensor, on themselves, their teams, patients, and the broader organization.

### Research Aims

#### Overview

Building on the NPT framework, this study investigated the key implementation challenges and facilitators for the novel Stay With It aftercare program from both patient and health care professional perspectives. We also examined whether an activity sensor is appropriate to support program implementation. We had 3 overarching research aims.

#### Research Aim 1: Challenges and Facilitators of Program Implementation at the Valens Rehabilitation Centre

First, we explored the challenges and facilitators that both patients and health care professionals encountered in implementing the Stay With It program at the Valens Rehabilitation Centre. We also assessed whether there were any unmet needs related to program implementation. This research aim was rooted in all 4 NPT constructs.

#### Research Aim 2: Perceived Usefulness of an Activity Sensor to Support Program Implementation and Sustainability

Second, we examined whether both patients and health care professionals found the activity sensor useful for program implementation. We also examined patients’ use of the sensor. This research aim was rooted in all 4 NPT constructs.

#### Research Aim 3: Maintenance of Physical Activity (Patient Adherence at Home)

Finally, we aimed to assess whether patients remained active during follow-up, patients’ self-reported achievement of home activity goals, and factors that influence daily physical activity at home. This research aim was specifically rooted in the NPT construct “reflexive monitoring.”

## Methods

### Ethical Considerations

The study was approved by the Cantonal Ethics Committee (Business Administration System for Ethics Committees [BASEC] number: 2021-02490) and preregistered on ClinicalTrials.gov (trial registration NCT05243407). All participants provided written informed consent.

### Study Design

We invited both patients participating in the Stay with It program at the Valens Rehabilitation Centre and health care professionals delivering the program to participate in this study. Data were collected between February 2022 and August 2022.

With respect to the patients, the study design was an observational longitudinal cohort study. Patients were enrolled when they started with the program and followed up for 3 months after being discharged. Study participation included the optional wearing of an activity sensor throughout the study and completion of qualitative and quantitative measures. Health care professionals were assessed in February and March 2022 before program implementation and again in August 2022 after they gained experience with the program.

### Study Participants

#### Patients

Adult inpatients (n=23) at the Valens Rehabilitation Centre who were undergoing rehabilitation and participating in the Stay With It program were eligible for the study. Those who were interested were provided with a study information sheet. The Stay With It program was designed for adults with either physical chronic health conditions or conditions at risk of becoming chronic, such as multiple sclerosis or cardiovascular disease. The program’s goal was to incorporate more physical activity into participants’ daily lives. Although patients were encouraged to wear an activity sensor throughout the study, this was not mandatory. We aimed to enroll approximately 20 patients in the study because this number was both feasible and would provide a variety of individual experiences.

#### Health Care Professionals

The participating health care professionals were occupational or physical therapists (n=13) at the Valens Rehabilitation Centre who were trained and scheduled to deliver the program.

### Stay With It Program

The Stay With It program, developed by the Valens Rehabilitation Centre, was designed to help patients integrate individualized activity and action plans into their daily routines once they return home. The program is divided into 4 sequential modules: “Introduction,” “Physical Strength,” “Endurance,” and “Application Into Daily Life.” A detailed description of the 4 modules is provided in Multimedia Appendix 1 [34,35].

### Study Procedure

#### Patients

**Figure 1:**
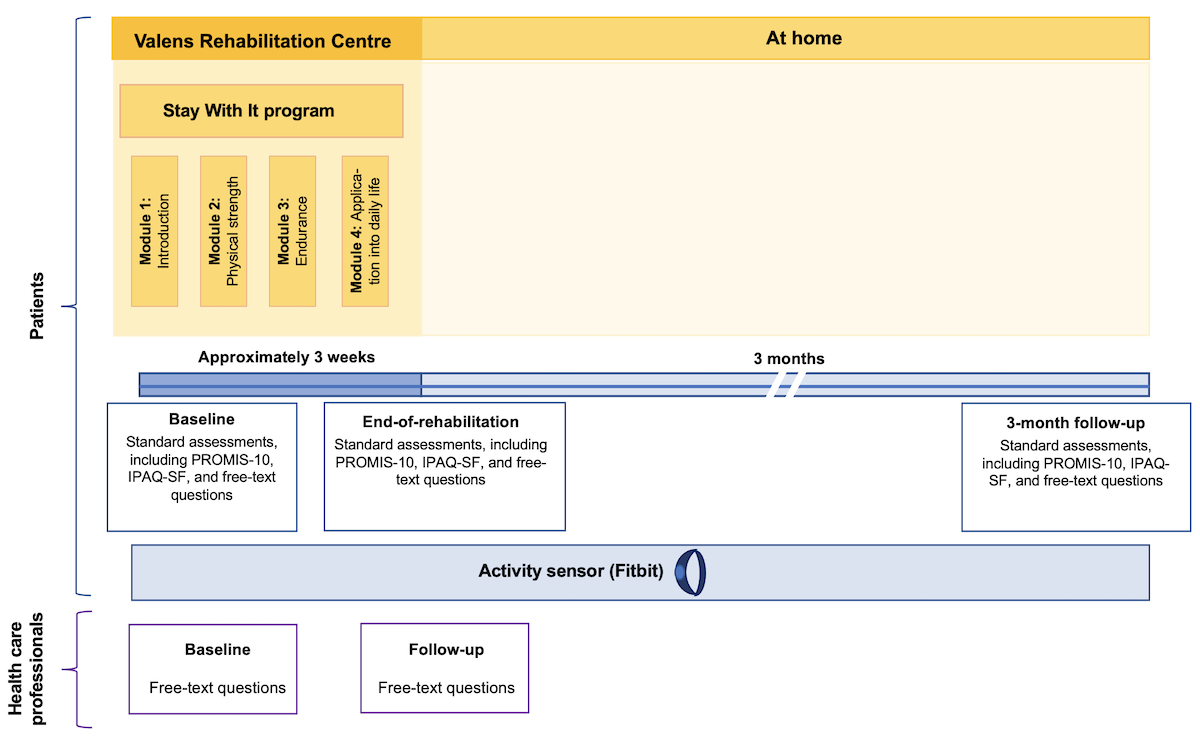
Visualization of the study procedure for patients and health care professionals. Patients participated in the Stay With It program during their rehabilitation stay at the Valens Rehabilitation Centre and were encouraged, but not required, to wear an activity sensor throughout the study. Their experience with the program, quality of life, and physical activity were assessed at baseline, the end of rehabilitation, and at a 3-month follow-up. Health care professionals were asked about their experience before implementing the program and again after having acquired experience with it. PROMIS-10: Patient-Reported Outcomes Measurement Information System–Global 10; IPAQ-SF: International Physical Activity Questionnaire–Short Form.

For patients, the study procedure consisted of five consecutive phases (Figure 1): (1) baseline session, (2) Stay With It program participation at the Valens Rehabilitation Centre, (3) end-of-rehabilitation assessment, (4) monitoring period at home, and (5) 3-month follow-up assessment. Daily-life activity data were continuously monitored throughout the study. If patients did not provide data for 5 days, the study staff checked to see whether they were having technical problems with their device, such as syncing issues. The 5 study phases are detailed in Textbox 1.

Daily-life activity data were assessed continuously from baseline until 3-month follow-up. Study personnel regularly checked the completeness of activity sensor data and reached out to participants if they did not provide any data for 5 days to check whether they were experiencing technical problems with their device (eg, device not synchronizing) and to offer technical support.

**Study phases for patients.**

1. Baseline session: after patients provided written informed consent, they were provided with a Fitbit Charge 4 (Fitbit LCC) activity sensor to be worn continuously on their nondominant wrist throughout the study. The study staff helped them set up the Fitbit app on their personal smartphone and connect their activity sensor to the app using a nonpersonal Fitbit study account. Patients were also given a brief introduction on how to use the app and what parameters were measured using the activity sensor. Patients then answered free-text questions about their motivation and expectations for the Stay With It program, as well as any prior experience with activity sensors. They also completed self-report questionnaires on quality of life and their daily physical activity over the previous week.
2. Stay With It program participation at the Valens Rehabilitation Centre: following the baseline session, patients took part in the Stay With It routine care program. Those with an activity sensor were encouraged to wear it continuously throughout their daily lives for monitoring to complement program participation.
3. End of rehabilitation: at the end of their rehabilitation stay, patients were phoned by the study staff and asked about their experience with the program and activity sensor and what activity goals they had set for their time at home. They were also readministered the self-report questionnaire on daily physical activity for the previous week.
4. Monitoring period at home: patients who had received an activity sensor were encouraged to continue wearing it during the 3-month monitoring period at home. The study staff routinely checked the completeness of these data.
5. Three-month follow-up: at the 3-month follow-up, the study staff contacted patients via phone. Patients were asked about their experiences and whether they felt that they had achieved their goals at home. They were also readministered questionnaires on quality of life and daily activity during the previous week.

#### Health Care Professionals

Health care professionals completed free-text questions before the program was implemented in February and March 2022 and again after they acquired experience with program implementation in August 2022.

### Measures

The measures of this study are described in detail in the subsequent sections.

#### Free-Text Questions

Both patients and health care professionals responded to free-text questions focusing on the challenges and facilitators of program implementation as well as the potential benefits of supporting program implementation using an activity sensor. These questions were developed in accordance with the 4 NPT constructs. Table 1 presents the 4 NPT constructs, along with brief definitions and descriptions of how they relate to the patients and health care professionals in this study.

From these core definitions, we developed free-text questions applying the 4 NPT constructs to this study. These questions, mapped to the 3 research objectives and the NPT constructs, are presented in Multimedia Appendices 2 and 3. As most patients experienced difficulties with fine motor skills, the study personnel (ZL and CH) assisted them in documenting their responses. Patients first dictated their responses, which the staff transcribed. The responses were then read back for verification and corrected as necessary.

#### Patient-Only Assessments

The following data and measures were assessed only in patients.

##### Daily-Life Activity–Sensing Data

For patients wearing an activity sensor, we assessed daily summed step counts and active and inactive minutes (highly active, fairly active, moderately active, and sedentary time).

##### International Physical Activity Questionnaire–Short Form

The International Physical Activity Questionnaire–Short Form is a 7-question retrospective self-report questionnaire with good validity for assessing daily physical activity across various intensities (high and moderate activity and walking) and time spent sitting for at least 10 minutes, averaged over the past 7 days [36].

##### Patient-Reported Outcomes Measurement Information System–Global 10

The Patient-Reported Outcomes Measurement Information System–Global 10 is a 10-item self-report instrument that assesses physical, mental, and social health as well as pain, fatigue, and quality of life [37]. This instrument was validated in multiple studies, and scores range from 0 (severe impairment) to 20 (optimal health) [37,38].

### Analytical Approach

Group comparisons for descriptive statistics were made using independent, 2-sided sample *t* tests (=.05) and were performed in R (version 4.3.1; R Core Team) using RStudio (version 2023.06.1; RStudio Inc). A detailed breakdown of how each of the free-text questions relates to the 3 research aims can be found in Multimedia Appendices 2 and 3.

#### Research Aim 1: Challenges and Facilitators of Program Implementation at the Valens Rehabilitation Centre

To identify the challenges and facilitators of program implementation, we used a thematic approach to analyze free-text responses from patients and health care professionals. The results are presented in a summary form, along with theme prevalence and exemplary, anonymized sample responses.

#### Research Aim 2: Perceived Usefulness of an Activity Sensor to Support Program Implementation and Sustainability

Consistent with the analytical approach used for research aim 1, we examined the free-text responses of patients and health care professionals using a thematic approach. Again, the results are presented in a summary form, along with theme prevalence and with exemplary, anonymized sample responses. We also visualized patients’ daily activity–sensing data using individual-level plots. Visualizations were created using the R package *ggplot 2*. We also determined descriptive statistics of daily-life activity as assessed by the activity sensor and the International Physical Activity Questionnaire–Short Form self-report questionnaire.

#### Research Aim 3: Maintenance of Physical Activity (Patient Adherence at Home)

The analytical approach for each of the 3 subcomponents of research aim 3 is outlined in the following sections.

##### Maintenance of Physical Activity Through Follow-Up

Given our limited sample size, we restricted our analysis to a visual examination of daily-life physical activity for overall trends, focusing on both intraindividual and interindividual variabilities.

##### Self-Reported Achievement of Home Activity Goals

We manually assessed the specificity of patients’ activity goal definitions using the SMART (specific, measurable, achievable, realistic, time-bound) criteria [34] and categorized them into 3 levels: high, moderate, and unspecific. Activity goals that met all the SMART criteria were considered highly specific. Activity goals that met between 1 and 4 of the SMART criteria were categorized as moderately specific. Goals that did not meet any of these criteria were considered unspecific. At 3-month follow-up, patients self-reported the degree to which they had achieved their activity goals. We classified goal attainment as “fully met,” “partially met,” or “not met” based on their self-reports.

##### Factors Influencing Daily Physical Activity at Home

We again used a thematic approach to examine free-text responses from patients at 3-month follow-up about what hindered and what facilitated physical activity in their daily lives at home.

## Results

### Sample Characterization

#### Patients

**Figure 2:**
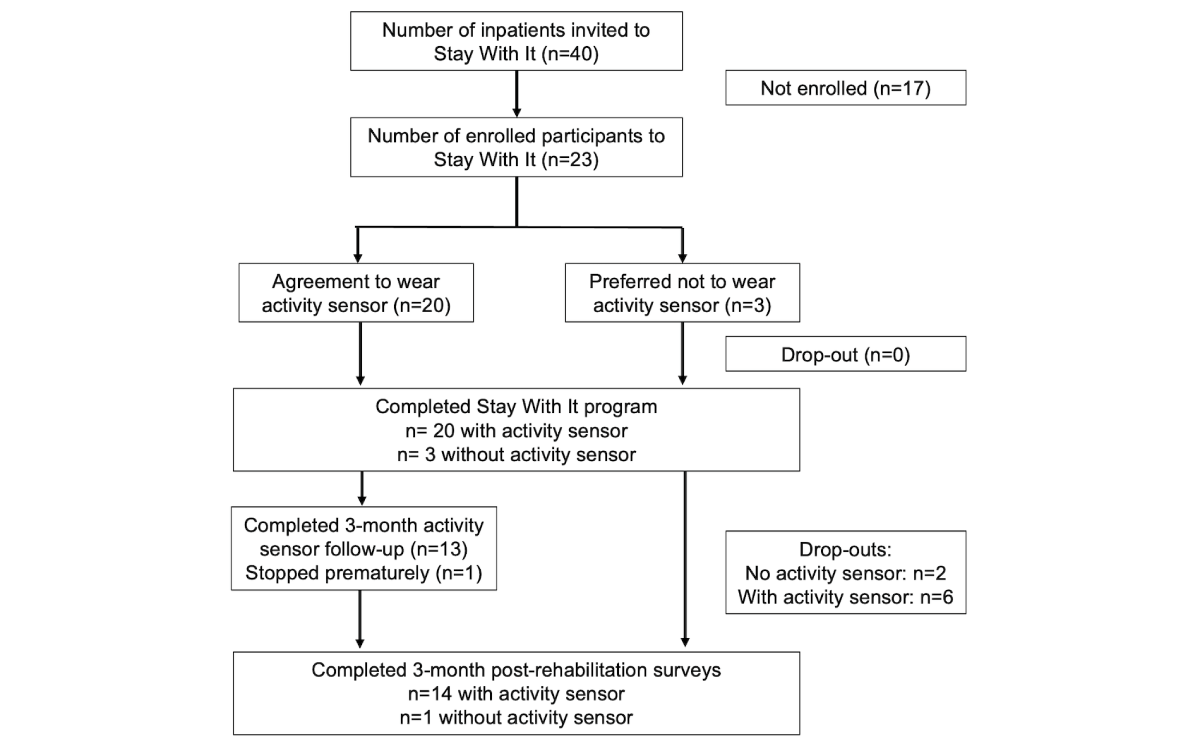
Study flowchart.

Patients’ age averaged 56.26 (SD 8.58; range 43-69) years, and 61% (14/23) were female. A characterization of patients with respect to their health conditions is provided in Multimedia Appendix 4. Of the 23 patients, 8 (35%) withdrew from the study early, of whom 6 (75%) were wearing an activity sensor. Of these 6 patients, 5 (83%) dropped out due to deterioration in their physical health, which made study participation frustrating (refer to Figure 2 for the study flow). The remaining patients who dropped out (3/8, 38%; with an activity sensor: 1/8, 13%; without an activity sensor: 2/8, 25%) were unreachable by phone at 3-month follow-up and thus did not provide any reasons for withdrawal. Descriptive statistics for study completers and noncompleters are provided in Table 2.

**Table 2:** Descriptive statistics at baseline (completers and noncompleters) and 3-month follow-up (completers).

Measure		Baseline				3-month follow-up
		Completer (15/23, 65%; with an activity sensor: 14/15, 93%; without an activity sensor: 1/15, 7%)	Noncompleter (8/23, 35%; with an activity sensor: 6/8, 75%; without an activity sensor: 2/8, 25%)	Mean difference (*P* value)	Completer (15/23, 65%; with an activity sensor: 14/15, 93%; without an activity sensor: 1/15, 7%)	
**PROMIS-10^a^, mean (SD)**						
	Physical health sum score	12.2 (2.54)	12 (3.16)	.89	13.92 (3.35)	
	Mental health sum score	12.93 (4.03)	11.83 (4.36)	.61	10.87 (1.46)	
**Activity sensor, mean (SD)**						
	Daily steps, mean (SD)	N/A^b^	N/A	N/A	At Valens: 8072.59 (3167.73); at home: 6853.71 (4542.79)	
**IPAQ-SF^c^(average minutes of activity intensity per day over the past 7 days), mean (SD)**						
	High intensity	92.14 (80.74)	46.61 (48.4)	.28	22.19 (23.33)	
	Moderate intensity	102.6 (90.63)	70.98 (63.17)	.29	42.34 (40.34)	
	Walking	51.62 (48.2)	29.2 (24.88)	.32	33.73 (48.16)	
	Sitting	338 (254.42)	363.75 (237)	.81	303.21 (171.95)	
**Attrition over the course of the study for patients who wore an activity sensor and patients who did not wear a sensor, n (%)**						
	Baseline: patients wearing an activity sensor (n=20)	N/A	N/A	N/A	Health deterioration: 5 (25); not available by phone: 1 (5)	
	Baseline: patients not wearing an activity sensor (n=3)	N/A	N/A	N/A	Not available by phone: 2 (67)	

^a^PROMIS-10: Patient-Reported Outcomes Measurement Information System–Global 10.

^b^N/A: not applicable.

^c^IPAQ-SF: International Physical Activity Questionnaire—Short Form.

#### Health Care Professionals

We assessed a total of 13 health care professionals, all of whom were occupational or physical therapists at the Valens Rehabilitation Centre and formed the team trained to deliver the program. Of the 13 health care professionals, 3 (23%) delivered all the program modules, whereas 10 (77%) delivered only a subset of the modules for organizational reasons.

### Research Aim 1: Challenges and Facilitators of Program Implementation at the Valens Rehabilitation Centre

Findings on challenges and facilitators are presented separately from the perspectives of patients and health care professionals, and the presentation is organized according to the 4 NPT constructs. The text detailing our findings is complemented by sample quotes.

#### Patient Perspective

##### Coherence

Before program participation, individuals reported a variety of challenges in attempting to maintain physical activity, which also sparked their interest in the program. On a personal level, many struggled with a lack of intrinsic motivation at home and physical limitations owing to pain, sensitivity to weather changes, and health issues (eg, falls). Outside the home, daily responsibilities; irregular working hours, especially for those who work from home; and the lack of therapeutic guidance and encouragement add to the burden (“Practicality at home is a challenge. In rehabilitation, there’s a set routine that’s often missing at home”). A person mentioned struggling with moving while others are watching, as this evokes feelings of shame.

##### Cognitive Participation and Collective Action

While at the Valens Rehabilitation Centre, patients had their rehabilitation training sessions integrated into their daily schedules to avoid conflicts with other appointments. The group program sessions (modules 2 through 4) were scheduled on Saturday mornings. On a few occasions, this conflicted with patients’ desire to visit their families. During their stay, primary care providers were informed of patients’ participation in the program to facilitate discussions about their program experience.

##### Reflexive Monitoring

Although the group setting was generally appreciated, of the 23 patients, 5 (22%) expressed a desire for a more homogeneous group composition, particularly in terms of both mobility and disease, to have more fruitful exchanges and avoid frustration. Moreover, among the 23 patients, 2 (9%) expressed a preference for more visual aids and fewer textual descriptions.

#### Health Care Professional Perspective

##### Coherence

Health care professionals had a good understanding of the program’s purpose before its implementation, as evidenced by their extensive knowledge of the barriers to physical activity when patients return home. Specifically, health care professionals identified the following reasons why patients have difficulty maintaining physical activity at home: lack of structure and support (6/13, 46%), difficulty maintaining motivation without constant motivational support from the health care team (6/13, 46%), responsibilities at home regaining priority (3/13, 23%), slipping back into old habits (2/13, 15%), lack of knowledge about how to apply the basic principles of physical activity (1/13, 8%), and difficulty creating an exercise plan at home that is both realistic and effective (1/13, 8%).

##### Cognitive Participation

Health care professionals identified several elements of the program that they expected to work well. Specifically, they (7/13, 54%) expected patients to share strategies for staying active with their peers in group discussions (“strategy sharing”). Some (2/13, 15%) also expected the program to provide patients with concrete guidance on how to incorporate and monitor exercise in their daily lives when they return home (“personalized plan”). Of the 13 health care professionals, 2 (15%) anticipated that the program would make patients aware of their current activity levels, which might lead them to find new ways to incorporate physical activity into their routines, such as taking the stairs more often (“awareness”), and 2 (15%) anticipated that the basic knowledge of the exercise principles documented in the program materials would help patients exercise more effectively at home (“expertise”).

Health care professionals also expressed some concerns about what might not work smoothly and need specific attention. The most common concern was that a long-term commitment without a structured environment or regular contact with their therapist may lead to a tendency to fall back into old patterns (“old patterns”; 4/13, 31%). Another concern was that for patients who are severely impaired, their limitations may make it difficult to find independent activities of daily living, and this might also be difficult to cope with emotionally. Patients who are less severely impaired should also not be overburdened to avoid demotivation (“demotivation”; 3/13, 23%). Finally, among the 13 health care professionals, 1 (8%) noted that it would be more realistic to plan activities of daily living that do not require extensive equipment or preparation to minimize time commitment (“feasibility”).

##### Collective Action

Health care professionals managed the logistical aspects of implementing the program, such as room reservations, weekly scheduling, and staffing for group sessions. The organization also integrated the program into Valens’ digital scheduling system to make it fit seamlessly into patients’ daily schedules. In addition, the process was integrated with the internal accounting system in consultation with the IT department.

##### Reflexive Monitoring

After having gained experience with the program, all (13/13, 100%) health care professionals were optimistic that a considerable number of patients would be able to incorporate and maintain more physical activity in their daily lives in the long term. Some (5/13, 38%) believed that the main factors determining whether patients would stay active were their internal motivation and the concreteness of their plan for returning home, such as outpatient exercise sessions. Among the 13 health care professionals, 1 (8%) also expected that, regardless of program participation, patients who were active before rehabilitation would be more likely to maintain activity at home than those with low activity levels before rehabilitation. A total of 2 (15%) health care professionals suggested that only patients who have the intention to be more active at home should be invited to participate in the program, as large differences in motivation negatively affect the group dynamic.

### Research Aim 2: Perceived Usefulness of an Activity Sensor to Support Program Implementation and Sustainability

Findings on the perceived potential of an activity sensor to support program implementation and sustainability are presented separately from the perspectives of patients and health care professionals, and the presentation is organized according to the 4 NPT constructs. The text detailing our findings is complemented by sample quotes.

#### Patient Perspective

##### Coherence

In terms of sense making, patients (20/23, 87%) who chose to use an activity sensor generally understood its potential to help them integrate program learning into their daily routines. Most (21/23, 91%) patients already used a smartphone and apps on a daily basis, and more than half (14/23, 61%) of them had previous experience with activity sensors.

##### Cognitive Participation

Of the 20 patients who chose to use an activity sensor, most had mostly positive expectations about using the activity sensor (n=17, 85%) and expected that it would give them control over their daily activity and remind them when they were not moving much (n=9, 45%). Among the 20 patients, 6 (30%) expected the activity sensor to motivate them to stay active, and 4 (20%) appreciated that the device could quantify movement (“The activity sensor helps me stay motivated”). Of the 23 patients recruited, 3 (13%) were open to trying the activity sensor, although they were unsure whether it would be useful for them. Some patients had concerns about using the device. Among the 23 patients, 2 (9%) expressed concerns about the technical operation of the device; 1 (4%) cited privacy issues; 1 (4%) felt that tracking their daily activity could reduce their enjoyment of the exercise; and 1 (4%) noted that devices such as watches often cause skin irritation, making direct wear on the body undesirable.

##### Collective Action

**Figure 3:**
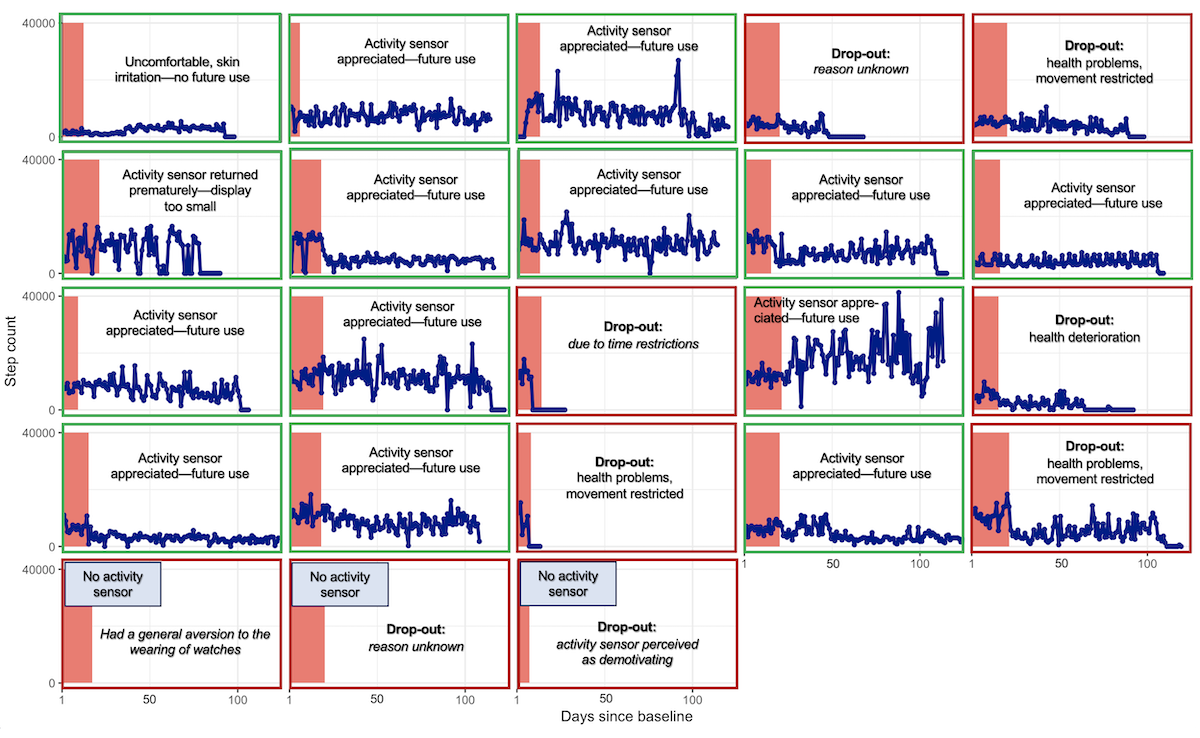
Individual-level time-series plots of daily step counts over the course of study participation.

**Figure 4:**
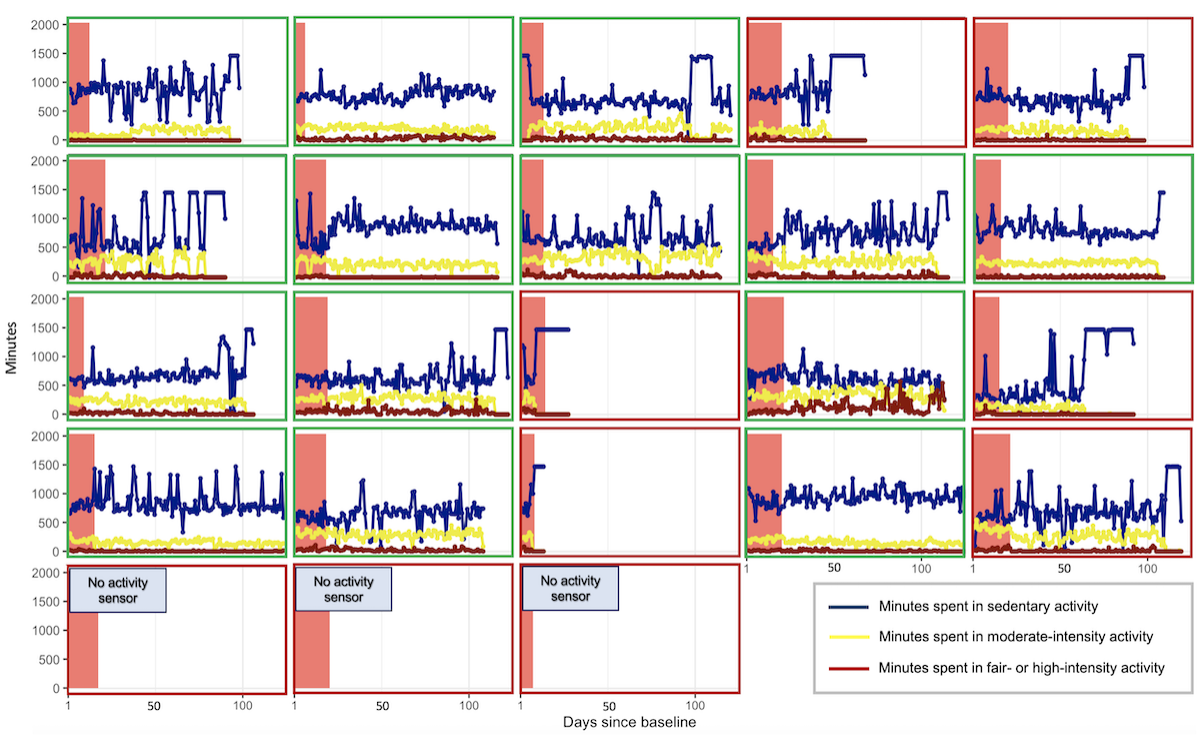
Individual-level time-series plots of raw minutes spent on different levels of daily activity.

Patients who chose an activity sensor and remained in the study (14/23, 61%) tended to wear the activity sensor continuously, as displayed in Figures 3 and 4.

##### Reflexive Monitoring

Of the 15 patients who were available for the 3-month follow-up, most (n=11, 73%) found the activity sensor to be an aid in staying physically active after returning home. A total of 10 (67%) patients planned to also wear an activity sensor in the future. Interestingly, patients who opted for an activity sensor and later dropped out of the study showed reduced sensor wear compliance just before dropout (refer to Figure 3). Baseline comparison between completers and noncompleters revealed no significant differences in self-reported average activity levels or quality of life (Table 2).

#### Health Care Professional Perspective

##### Coherence

Before the launch of the program, all (13/13, 100%) health care professionals were optimistic about using activity sensors to support the program. Of the 13 health care professionals, 3 (23%) viewed the sensor primarily as a motivational tool, whereas 2 (15%) believed that its main benefit would be to quantify daily exercise (“An activity sensor simplifies quantifying movement and activity”). Some concerns were raised, such as that some patients may not be technically adept enough (4/13, 31%) and the possibility of demotivation if the sensor showed unmet goals (“Failure to achieve goals can lead to frustration”). In addition, 2 (15%) of the 13 health care professionals were concerned that patients with fine motor difficulties might have difficulty wearing or removing the sensor.

##### Cognitive Participation and Collective Action

Before the start of the study, all (13/13, 100%) health care professionals received a sample of the activity sensor used in the study and performed a test run with it. This ensured that everyone was familiar with the specific type of activity sensor.

##### Reflexive Monitoring

During program implementation, health care professionals identified motivation and self-monitoring (both 7/13, 54%) as the most valuable aspects for patients. However, they also identified challenges. Among the 13 health care professionals, 6 (46%) expressed that some patients felt frustrated when comparing themselves with others or when not meeting their (at times unrealistic) goals. Moreover, 3 (23%) health care professionals reported that the sensor’s measurements were occasionally inaccurate with certain physical impairments or when patients used assistive devices such as walkers or canes. A total of 3 (23%) health care professionals also observed that some patients had difficulties with the technical aspects of the sensor.

### Research Aim 3: Maintenance of Physical Activity (Patient Adherence at Home)

#### Maintenance of Physical Activity Through Follow-Up (Collective Action)

The levels of physical activity over the 3-month follow-up period varied considerably, both within and between patients, as displayed in Figures 3 and 4.

#### Self-Reported Achievement of Home Activity Goals (Collective Action)

As part of the final Stay With It session, patients were guided to define 3 activity goals for their time back at home that they wished to pursue. In the event that the patients missed this session and thus did not set goals as part of the program, they were asked whether they had any self-defined goals for their time back home, and all of them did. One participant could not be reached by phone for the end-of-rehabilitation assessment, resulting in activity goals for 22 patients. The patients in our study defined, on average, 2.60 (SD 0.68; range 1-3) goals for their time back home, resulting in a total sample of 51 activity goals. On the basis of the SMART criteria, the majority of goals were of moderate specificity (20/51, 39%), whereas 31% (16/51) were highly specific, and 29% (15/51) were unspecific. At 3-month follow-up, patients self-reported their achievement of these goals, the results of which are detailed in Table 3.

Highly and moderately specific goals were reported as fully achieved in 53% (19/36) of the goals, whereas only 20% (3/15) of the nonspecific activity goals were reported as fully achieved. For 47% (7/15) of the nonspecific goals, goal achievement could not be assessed because the respective participants had dropped out before the 3-month follow-up. For highly or moderately specific goals, this was the case for 19% (7/36) of the activity goals.

**Table 3:** Activity goals of varying precision of the patients who completed the 3-month follow-up assessment^a^.

Specificity of activity goals (N=51 goals)	Anonymized examples for activity goals^b^	Self-reported goal achievement at 3-month follow-up, n (%)
Highly specific goals (n=16, 31%)	“10,000 steps per day” “At least 6 hours sleep per night” “Going outside for a walk twice a week. Instead, I do less of the housework, or I do it with less perfection” “Climbing 6 to 10 floors a week” “Working out 3 times a week for about 30 minutes each session”	Fully: 8 (50) Partially: 3 (19) Not achieved: 1 (6) Missing information because of participant dropouts: 4 (25)
Moderately specific goals (n=20, 39%)	“I would like to work out less but more often (ie, 2 to 3 times a week). I plan to use resistance bands to build muscle strength.” “I want to do more stretching. I also plan to go to physical therapy once a week for instructed stretching.” “Cycling/walking as far as possible” “Going to the gym again, that I exercise more, doing something every day—be it mowing the lawn, cleaning the windows or something else. It doesn’t really matter.”	Fully: 11 (55) Partially: 2 (10) Not achieved: 4 (20) Missing information because of participant dropouts: 3 (15)
Unspecific goals (n=15, 29%)	“I want to adapt my fitness program in a way that is not overwhelming, but moderate.” “My goal is to become more fit.” “I’m aiming to incorporate some form of exercising into my daily life.” “I want to keep on going, to keep on overcoming, to keep on participating.”	Fully: 3 (20) Partially: 2 (13) Not achieved: 3 (20) Missing information because of participant dropouts; 7 (47)

^a^Because of rounding, percentages may not add to 100%.

^b^Translated and edited for privacy.

#### Factors Influencing Daily Physical Activity at Home (Reflexive Monitoring)

In terms of reasons for not achieving the activity goals, the mentioned barriers to physical activity included the heat in summer or being sensitive to the weather in general (5/15, 33%), as this leads to reduced energy levels and fatigue. Pain and deterioration in physical health (3/15, 20%), for example, due to an illness or accident, were also common barriers. Distraction from potentially time-consuming indoor activities, such as the use of the internet, was also cited as a barrier to physical activity (2/15, 13%). The activity sensor was mentioned because it was frustrating for patients to see how little they were moving when they experienced barriers or because steps were not counted when using a rollator. In terms of facilitators for staying active, 73% (11/15) of the patients found the activity sensor helpful, 46% (7/15) mentioned regular exercise routines (eg, physical therapy) and group dance or exercise classes as helpful, 33% (5/15) had a partner who participated in their physical activity, and 13% (2/15) benefited from having a dog to help them stay active.

## Discussion

### Principal Findings

Using the NPT framework, this study examined the challenges and facilitators of implementing an aftercare program into routine care and whether activity sensors could support program implementation. Over a 3-month follow-up period, we examined several aspects of patients’ physical activity. We found that the consideration of both patient and health care professional perspectives is critical to the successful implementation of complex interventions. In addition, there was considerable variability in patients’ activity levels and goal attainment at home, influenced by multiple limiting factors.

Our findings for research aim 1 add to previous implementation science research emphasizing the need to consider both patient and health care professional perspectives when integrating complex interventions into routine care (eg, the study by Naef et al [39]). Patients in our study faced various barriers to daily physical activity but were committed to increasing activity after rehabilitation. Health care professionals saw the potential of the program and believed that success depended on individual drive and a robust postrehabilitation plan. The main challenges in implementing the program were organizational, such as integrating the program into the scheduling and accounting system, which was essential for its seamless integration into routine care. In addition, the importance of appropriate timing was highlighted by the occasional conflict between patients’ personal commitments and weekend group sessions. Our findings resonate well with a 2021 review that examined exercise adherence in patients with chronic conditions and older adults [7]. The review highlighted 14 key factors, with the most relevant factors to our research being the use of technology, the initial assessment of participant characteristics, challenges and facilitators, participant education, clear expectations, and goal setting.

As for the perceived usefulness of an activity sensor in supporting the implementation and sustainability of the Stay With It program (research aim 2), most patients who used the activity sensor appreciated monitoring and “objectifying” their daily activity. Consistent with this finding, patients who opted to wear an activity tracker at baseline had high compliance rates for daily wear time. This conclusion aligns with a 2020 systematic review and meta-analysis suggesting that activity sensors may be a useful tool in promoting active lifestyles in patients [40]. However, our results also suggest that the perceived usefulness of an activity sensor may vary depending on individual situations, even within the same individual. In our study, 35% (8/23) of the patients dropped out prematurely. Our results align with a recent mobile health study of chronic low back pain with a 6-month follow-up [41]. This study compared an intervention group that received a face-to-face home visit, 12 telephone sessions, and an activity sensor with a control group that received only physical activity information and advice to stay active. The intervention group had a dropout rate of 9%, whereas the control group had a dropout rate of 42%. Although the intervention group was equipped with an activity tracker, they also benefited from ongoing personal support. This highlights the potential influence of personal engagement and a lack of support as factors that may have played a key role in preventing dropout. Notably, in our study, of the 8 patients who dropped out, 7 (88%) had previously indicated that declining physical health made monitoring daily activity burdensome and believed that discussing their minimal progress could negatively affect their mental well-being. Interestingly, patients tended to stop wearing the activity sensor just before withdrawal. This potentially transitional period suggests an opportune time for targeted interventions to increase support for participants to achieve their goals. Future research would benefit from providing tailored support to inspire individuals to reintegrate physical activity and regain motivation after a decline, for example, through proactive telephone outreach. An example of achieving high compliance using proactive telephone outreach is a recent study by Sieber et al [28]. This longitudinal activity-sensing study examined people with multiple sclerosis, consisted of a 2- to 3-week inpatient stay and then a 1-month follow-up, and reported a dropout rate of only 4% [28].

Regarding research aim 3, our study found that patients exhibited a wide range of activity levels after returning home, both on an individual basis and in comparison with each other. In addition, the self-reported achievement of home activity goals varied. In addition, successful monitoring of self-reported home activity goals was complicated by the fact that a number of these goals did not meet the SMART criteria set. Interestingly, although a subset of patients with vague goals missed the final Stay With It module, which guided patients in setting activity goals for their time at home, leading them to set activity goals for their return home autonomously, ambiguities in goal setting persisted even among patients who completed the module. This underscores that although group settings can promote the sharing of ideas and increase motivation, personalized guidance and direct collaboration with health care professionals may be more effective for some patients in defining realistic and motivating goals. In terms of barriers to physical activity at home, we found that patients often cited fatigue or decreased energy due to weather, especially heat, in addition to pain and declining physical health. This aligns well with a comprehensive 2017 narrative review on multiple sclerosis, which identified issues such as disease-related limitations; personal beliefs; fatigue; the lack of understanding the benefits of exercise; and practical hurdles, including financial issues, the lack of support, and the lack of accessibility, as key barriers to physical activity [42].

This study has some limitations that need to be considered. Our study was exploratory and observational in nature, with a limited sample size. Patients were also recruited from the same study site, and health care professionals invited those they felt would benefit from the program. It is likely that this led to a selection bias, with a preference for patients who were already inclined to incorporate more physical activity into their daily lives. It is also difficult to disentangle the relative effects of the program and the activity sensors. Previous research has also shown that simply wearing an activity sensor can promote a healthier lifestyle by encouraging self-monitoring [40,43]. To truly distinguish between the individual effects of the program and activity sensors, future research would need to include control groups. For example, in a subsequent study, 1 group could be assigned to receive the Stay With It program in combination with an activity tracker, whereas a control group could receive only the activity tracker. Ideally, a third group would receive standard care, such as that offered in Valens, without the activity tracker, which would further help in assessing the effect. Because not using a sensor also precludes the collection of daily-life data, an alternative could be a blinded activity sensor with the display covered (eg, by tape). This approach was successfully used in the 2020 study by Bentley et al [44].

Consumer-grade activity sensors are affordable and easy to use, but their accuracy can be limited for patients with physical impairments. For example, patients with Parkinson disease often have shorter, shuffling steps and slower walking speeds (for an overview, refer to the study by Wendel et al [45]). Because many of the step-counting algorithms in such sensors are based on data from healthy individuals, their accuracy may be reduced for unique movement patterns. Slower speeds may not trigger the algorithm to detect the steps. This observation is consistent with the findings that consumer-grade sensors show reduced accuracy in detecting shorter stride lengths, particularly in patients with musculoskeletal and neuromuscular conditions in a rehabilitation setting [41].

In terms of lessons learned, the findings from our study indicate that patients perceive a benefit from preparing for their return home in a detailed fashion, and the group setting was generally well received. This approach is particularly effective in settings where groups include patients with similar impairments and motivations, facilitating a mutual exchange of ideas. However, challenges arise when patients vary widely in their health conditions, as such disparities can create tensions: patients who are more severely impaired may feel frustrated when confronted with the severity of their disease, whereas patients who are less impaired may not only miss out on benefits but also feel anxious about their future health trajectory. Another lesson learned is that some patients have difficulty setting specific, realistic activity goals. In addition to being measurable, these goals also need to be motivating, as overly ambitious activity goals can lead to a constant sense of failure. In some cases, activity goals may be best defined in a one-on-one setting rather than in a group setting. Another key lesson from our research is the importance of involving health care professionals in the implementation process. In our study, these professionals were adept at identifying organizational shortcomings and were attuned to group dynamics. However, overburdening them with additional tasks is likely to be a hindrance to implementation and, in the long run, may drain their commitment. The study team assisted with setting up the activity sensor and documenting responses to free-text questions, which may have increased patient motivation. In future implementations, ongoing communication between patients and their primary therapists regarding the program and the activity sensor may continuously strengthen their motivation.

### Conclusions

In conclusion, our research shows that integrating aftercare programs into routine care holds promise for supporting patients to stay active at home. Activity sensors may facilitate these efforts, although their potential may vary depending on individual health status, the level of impairment, and personal preferences. In particular, remote physical activity monitoring can also open new avenues for postrehabilitation care by signaling windows for timely interventions, for instance, through motivational messages in the event of a physical activity decline. Finally, our research also highlights that considering health care professionals’ perspective is critical when implementing novel measurement devices (such as activity sensors) and novel interventions into routine care.

## Appendix

Multimedia Appendix 1 Overview of the 4 modules from the Valens Rehabilitation Centre’s Stay With It program.

Multimedia Appendix 2 Free-text questions posed to patients and how they correspond to the 4 normalization process theory constructs and the study’s 3 overarching research aims.

Multimedia Appendix 3 Free-text questions posed to health care professionals and how they correspond to the 4 normalization process theory constructs and the study’s 3 overarching research aims.

Multimedia Appendix 4 Health conditions of patients participating in the study.

## Abbreviations

BASEC: Business Administration System for Ethics Committees
NPT: normalization process theory
SMART: specific, measurable, achievable, realistic, time-bound
