# Supplementary material for: Implementation of Remote Activity Sensing to Support a Rehabilitation Aftercare Program: Observational Mixed Methods Study With Patients and Health Care Professionals
Source: JMIR Mhealth Uhealth. 2023 Dec 8;11:e50729. doi: 10.2196/50729 (PMC10746974; doi:10.2196/50729)
Supplement: Multimedia Appendix 1 [file mhealth_v11i1e50729_app1.pdf]

Overview of the Four Modules from Valens Rehabilitation Centre's 'Stay With It' Program

'Introduction' module. The introductory module of the program is conducted on a one-on-one basis between the health professional and the participant and begins with some basic information about the benefits of physical activity, which is discussed interactively and supported by a short text section on physical activity. The module then continues with a series of open-ended questions that are listed in the booklet with space for participants to make notes for later. The questions focus on how often and in what ways the participant usually moves or exercises at home (e.g., 'How often do you move in your daily life?'), what kinds of physical activities they appreciate or enjoy during rehabilitation (e.g., 'What physical activities did you particularly like or enjoy during your rehabilitation stay?'), ideas for exercise at home and whether there are activities they used to enjoy that they no longer do or have always wanted to try. The program also uses motivational interviewing techniques and asks participants to think about the advantages and disadvantages of incorporating more physical activity into their daily lives at home (e.g., 'What are the long-term benefits of incorporating more physical activity into your daily routine?'). Advantages might include increased physical fitness and the cons might include a time commitment. The module concludes by asking participants to consider how much physical activity they would realistically like to do when they return home.

'Physical strength' module. This module took place in a group setting and began with general information about physical strength, with an emphasis on the different types of activities that participants might engage in. Participants discussed a series of open-ended, interrelated questions in the group, exploring a list of different activities of varying intensity (e.g., going for a walk, cooking, doing housework, aerobic, gardening) to see what they have done and appreciated in the past and what would be of interest for their time back home. The module also focused on concrete planning ('When and how often do you see yourself doing these activities?') and feasibility in terms of whether individuals would be motivated to do activities alone or if they could ask their partner or a friend to do them with them ('If you

don't want to do the training alone, who could you motivate to join you?'). The module concluded with a collection of ideas on how to put the plans into practice (e.g., joining a sports club, creating a personal training routine, etc.).

'Endurance' module. The endurance module also took place in a small group setting and focused more on the regularity of physical activities of different activities in their daily lives. The module also introduced the BORG scale [35], which provides a classification for physical activity that is close and applicable to the individual's daily life. On a scale from 0 to 10, participants rated the intensity of a given activity, where 0 corresponds to 'no effort' and 10 corresponds to 'maximum effort'. Participants were then encouraged to count weekly minutes of activity and assign them to the BORG scale. They then discussed a list of suggested activities that may be appropriate to increase their weekly efforts to be more active. The module concluded with a discussion of how participants can most efficiently organize and integrate activities into their daily lives.

'Application into daily life' module. The final module, also conducted in small groups, focused on the transition of the learning into daily life. The participants first discussed how they could concretely approach the integration of the newly learned material and what specific goals they had for their time back home. Each person was asked to define three specific goals for their return home. Participants were guided to define their goals so that they would meet the SMART criteria (i.e., Specific, Measurable, Achievable, Realistic, Time-Bound) [34] and were encouraged to think about how they could later measure what they had set out to do. Participants then rated their confidence in incorporating the planned activity into their daily lives on a scale of 1 (not at all) to 10 (very much). For example, they rated whether they were likely to participate in endurance training ('Do strength training three times a week') and be more active at home ('More exercise at home'). At the end of the module, in order to give their plans as much detail as possible, the participants compiled a weekly activity plan.
