# Supplementary material for: Implementation of Remote Activity Sensing to Support a Rehabilitation Aftercare Program: Observational Mixed Methods Study With Patients and Health Care Professionals
Source: JMIR Mhealth Uhealth. 2023 Dec 8;11:e50729. doi: 10.2196/50729 (PMC10746974; doi:10.2196/50729)
Supplement: Multimedia Appendix 2 [file mhealth_v11i1e50729_app2.pdf]

Free-text questions posed to patients and how they correspond to the four Normalization Processing Theory (NPT) constructs and the study's three overarching research aims

**Baseline (prior to program participation)**		**End-of-rehabilitation**		**3-month follow-up**	
Free-text questions	Corresponding Research Aim	Free-text questions	Corresponding Research Aim	Free-text questions	Corresponding Research Aim
**NPT construct: Coherence**					
What makes it hard for you to be active at home?	Research Aim 1	*Only if the module has been accomplished:* What do you gain from the module 'Introduction' of the 'Stay with it' program?	Information for later internal revision of program materials	-	
What helps you to be active at home?	Research Aim 1	*Only if the module has been accomplished:* What do you gain from	Information for later internal revision of	-	

		the module 'Physical strength' of the 'Stay with it' program?	program materials		
What is your motivation to participate in the program 'Stay with it'?	Research Aim 1	*Only if the module has been accomplished:* What do you gain from the module 'Endurance' of the 'Stay with it' program?	Information for later internal revision of program materials	-	
		Only if the module has been accomplished: What do you gain from the module 'Everyday life' of the 'Stay with it' program?	Information for later internal revision of program materials		
**NPT construct: Cognitive participation**					

Can you imagine wearing an activity tracker as part of the 'Stay with it' program? If yes, please briefly describe how it could support you.	Research Aim 2			*If Fitbit has been worn:* What was your experience with the Fitbit? Has it supported you in reaching your activity goals?	Research Aim 3
Do you have concerns of any kind regarding the use of activity trackers? If yes, what are they?	Research Aim 3	Is there anything else you would have needed?	Research Aim 1	Did you succeed in being active at home? *Follow-up questions to elicit further details if patient response lacks detail:* What worked well? What was difficult?	Research Aim 3
**NPT construct: Collective action**					

Have you ever used an activity sensor like the Fitbit before? If so, what was your experience?	Research Aim 2	*If Fitbit has been worn:* Do you plan to wear the Fitbit regularly at home?	Research Aim 2	What helped you stay active at home?	Research Aim 3
Do you regularly use your smartphone in your daily life? *Follow-up question to elicit further details if patient response lacks detail:* Do you use apps frequently?	Research Aim 2	*If Fitbit has been worn:* What was your experience with the Fitbit? *Follow-up question to elicit further details if patient response lacks detail:* Did it support you in reaching your activity goals?	Research Aim 2	What made it difficult for you to be active at home?	Research Aim 3
**NPT construct: Reflexive monitoring**					

-		As part of ‘Stay with it’, what 3 specific goals have you defined?<br>Goal 1:<br>Goal 2:<br>Goal 3:<br><br>*For each goal, if patient wore an activity sensor:*<br>How can the goal be verified using the Fitbit?	Research Aim 3	Has participating in the 'Stay with it' program been helpful to you?	Information for later internal revision of program materials
		Do you have any requests or suggestions for future ‘Stay with it’ implementations?	Information for later internal revision of program materials	*If Fitbit has been worn:*<br>Can you imagine using an activity sensor again in the future?	Research Aim 2

		Additional remarks	Information for later internal revision of program materials	Additional remarks	Information for later internal revision of program materials
