# Supplementary material for: Implementation of Remote Activity Sensing to Support a Rehabilitation Aftercare Program: Observational Mixed Methods Study With Patients and Health Care Professionals
Source: JMIR Mhealth Uhealth. 2023 Dec 8;11:e50729. doi: 10.2196/50729 (PMC10746974; doi:10.2196/50729)
Supplement: Multimedia Appendix 4 [file mhealth_v11i1e50729_app4.pdf]

**Health condition**	**N (%)**
Multiple sclerosis	12 (52.17%)
Lumbar spondylogenic (pseudoradicular) syndrome and radicular pain syndrome	4 (17.39%)
Parkinson’s disease	2 (8.70%)
Chronic panvertebral syndrome	1 (4.35%)
Spasticity of the lower extremities due to cerebral palsy	1 (4.35%)
Infection and inflammatory reaction due to internal prostheses, implants or grafts in the nervous system	1 (4.35%)
Unspecified flaccid paraparesis and paraplegia	1 (4.35%)
Complex regional pain syndrome	1 (4.35%)
